# Antimicrobial resistance and rational prescription practices: knowledge, perceptions and confidence of health profession interns in Uganda

**DOI:** 10.1093/jacamr/dlad105

**Published:** 2023-10-03

**Authors:** Shamim Nabidda, Rogers Ssennyonjo, Joseph Atwaru, Andrew Marvin Kanyike, Shiellah Baryayaka, Kennedy Pangholi, Jonans Tusiimire

**Affiliations:** Faculty of Medicine, Mbarara University of Science and Technology, Mbarara, Uganda; Aga Khan University, Kampala, Uganda; Faculty of Health Sciences, Busitema University, Mbale, Uganda; Faculty of Medicine, Mbarara University of Science and Technology, Mbarara, Uganda; Faculty of Health Sciences, Busitema University, Mbale, Uganda; Department of Internal Medicine, Mengo Hospital, Kampala, Uganda; Faculty of Medicine, Mbarara University of Science and Technology, Mbarara, Uganda; Faculty of Health Sciences, Busitema University, Mbale, Uganda; Faculty of Medicine, Mbarara University of Science and Technology, Mbarara, Uganda

## Abstract

**Background:**

Antimicrobial resistance (AMR) is significantly driven by misuse and overuse of antibiotics. Graduate health profession interns often prescribe antimicrobials under minimum supervision.

**Objectives:**

This study explored the knowledge, perceptions and confidence of health profession interns in Uganda regarding AMR and rational prescription practices.

**Methods:**

This was a cross-sectional survey employing quantitative techniques carried out between October and November 2022 at six tertiary hospitals in Uganda. Health profession interns including doctors, nurses, midwives and pharmacists were recruited as study participants. Data were collected using online Kobo toolbox software. Data analysis was performed using STATA (StataCorp) version 16. Bivariate analysis and multivariable logistic regression were performed. *P* < 0.05 was considered statistically significant.

**Results:**

We recruited 281 participants with a mean age of 27 ± 3.8 years, of which few (*n* = 53; 19%) had good knowledge about AMR and rational prescription. The use of professional organization guidelines as a source of information was significantly associated with good knowledge (adjusted OR = 1.9; 95% CI: 1.0–3.5; *P* = 0.046). Nurses had the least knowledge compared with doctors and pharmacists. Continuous medical education (99%) and availability of clinical guidelines (98%) were identified as the most helpful intervention to improve knowledge. Most participants were confident about accurately diagnosing infections and sepsis and selecting appropriate antimicrobials.

**Conclusions:**

Continuous medical education and availability of clinical and professional organization guidelines should be leveraged to improve the knowledge of AMR and rational prescription among health profession interns. Their high confidence in rational prescription practices should be pivotal to the fight against AMR.

## Introduction

Antimicrobial resistance (AMR) is a situation where microorganisms evolve to become resistant to treatment to which they were previously susceptible, leading to treatment failure, severe illness and even death.^1^ The misuse and overuse of antibiotics has escalated worldwide spread of antimicrobial-resistant organisms, which is a big threat to global health.^2,3^ It is projected that by 2050 AMR will lead to the death of up to 10 million people, especially in developing countries with a great burden of severe and life-threatening infections.^1,4^ The 2015 Uganda situation analysis report on AMR showed that microbial infections like pneumonia, sepsis and TB accounted for about 18.4% of hospital-based mortality yet resistance to the most commonly used antimicrobials (e.g. penicillins, tetracyclines, co-trimoxazole) was above 80% in some cases, with a high prevalence of MDR bacteria such as MRSA and ESBL producers.^5^

Despite the fact that AMR develops naturally, it is escalated by irrational use of antimicrobials due to factors like poor prescription practices and the inadequate health workers’ knowledge on AMR.^6,7^ Prescribers therefore have a vital role in the fight against AMR through their rational and safe prescription practices.^8^ Although in some healthcare systems, physicians and senior doctors are responsible for prescribing antibiotics because the junior doctors are deemed to have limited knowledge and skills,^9^ the prescribing decision is often left to junior doctors in most settings.^3,10^ Topics on AMR are included in medical schools’ curricula worldwide, but the knowledge and confidence of newly graduated health profession interns is uncertain.^11^

Indeed, in several studies, irrespective of the fact that junior doctors have proved to be aware of AMR,^12–14^ they make relatively many prescribing errors due to lack of prescribing competencies, possibly due to inadequate undergraduate education and training in clinical pharmacology and therapeutics.^10^ Most of these junior doctors select antimicrobials based on the practice of their older colleagues, who may be deficient in some prescription areas, rather than on official guidelines. Therefore, it is important that they have a comprehensive knowledge regarding drugs, including antimicrobials to improve the judicious use of antimicrobials.^2^

In Uganda, health profession interns of different cadres including doctors, nurses and pharmacists are posted in different tertiary and general hospitals across the country for 1 year training under the supervision of specialists and consultants before they are certified by respective bodies to practise independently. These junior health professionals constitute a great part of the health work force and sometimes have to work under minimum supervision, therefore it is imperative that they are best equipped with good knowledge about AMR and good prescription practices. However, little is known about this important matter in Uganda. This study explored the knowledge, perceptions and confidence of health profession interns at six tertiary hospitals in Uganda regarding AMR and rational prescription practices.

## Materials and methods

### Study design

This was a descriptive cross-sectional, multicentre survey employing quantitative techniques carried out between October and November 2022.

### Study setting

The study was carried out at six tertiary hospitals at the highest level of health system hierarchy in Uganda called referral hospitals, which also double as teaching and training hospitals for health profession interns with the required specialists and most antimicrobial drugs. They included: Mbarara Regional Referral Hospital in Southwestern Uganda, serving approximately 3.6 million people; Mbale Regional Referral Hospital in the East, serving about 4.7 million people; Gulu Regional Referral Hospital in the North; and Mulago National Referral Hospital and Kiruddu and Kawempe General Hospitals, all part of the Mulago National Referral Complex.

### Study population

We recruited health profession interns including doctors, nurses, midwives and pharmacists at the above-mentioned hospitals. The sample size was calculated using Epi Info StatCalc for population surveys. Using a study prevalence percentage (56%) of good knowledge on AMR by junior doctors,^14^ acceptable margin error of 5% and design effect of 1.0 at 95% CI, the calculated sample size was 289. A proportional sample of representative interns from each hospital depending on the total number was calculated and selected as shown in Table 1.

**Table 1.** Calculated sample size proportion for the different study sites

Hospital	Estimated population	Sample
Mbale Regional Referral Hospital	57	50
Mbarara Regional Referral Hospital	33	30
Mulago National Referral Hospital	105	82
Gulu Regional Referral Hospital	30	28
Kiruddu General Hospital	57	50
Kawempe General Hospital	56	49
Total	338	289

### Data collection and sampling procedure

For each participating site, a research assistant was identified and trained on the unique study aspects. For each internship site, names of participants were randomly chosen from the available lists considering total numbers as allocated from sample size calculations. Data were collected using online Kobo toolbox software shared by research assistants to the selected participants. The research assistants physically approached the selected participants with the consent form and upon acceptance they shared with them the data collection tool link via their WhatsApp inbox or e-mail address.

### Data collection tool

The data collection tool was adopted from a validated tool from a similar study^14^ and modified to suit our study. The questionnaire was pre-tested among health profession interns who were not to be part of the study participants to ensure its comprehensible. It consisted of 40 questions aggregated in five parts. The first part, Part A, collected demographic information such as sex, hospital, cadre of medical intern’s prior qualification and training on rational prescription, among others. Part B collected the participant’s knowledge about AMR and rational prescription, assessed using six questions, of which three questions were clinical vignettes, one question was about the situation of AMR in Uganda obtained from the Antimicrobial Resistance National Action Plan document, which is publicly accessible, and two questions from WHO/INRUD core prescribing indicators, respectively. The questions were answered in a ‘Yes’, ‘No’ or ‘Not sure’ format. Each correctly chosen choice had a value of 1, and each wrongly chosen or ‘Not sure’ response had a value of 0. Participants’ overall knowledge was categorized, using the modified Bloom’s cut-off point, as good if the score was between 80% and 100%, moderate if the score was between 50% and 79%, and poor if the score was less than 50%. Part C consisted of perceptions of health profession interns on the causes of AMR, with six statements answered on a 5-point Likert scale from strongly agree to strongly disagree. Part D entailed 11 statements about perceptions on the potential helpful interventions to improve antimicrobial prescribing, answered on a 5-point Likert scale from very helpful to very unhelpful. For the last nine questions in Part E, they assessed confidence level of prescription, answered on a 5-point Likert scale from very confident to very unconfident.

### Data analysis

The data were extracted from the Kobo toolbox software into Microsoft Excel 2016 (Microsoft Corporation) for cleaning and coding. The data were then exported to STATA (StataCorp) version 16 for further analysis. Numerical data were tested for normality and summarized as either means and standard deviations or median and ranges. Categorical data were summarized as frequencies and proportions and presented in tables and graphs. Associations between independent and dependent variables were assessed at bivariate analysis using chi-squared or Fisher’s exact test, one-way ANOVA and independent *t*-test. Those variables with a *P* value of less than 0.2 at bivariate analysis and those with biological significance were considered for multivariable logistic regression. *P* < 0.05 was considered statistically significant.

### Ethics

The study was conducted according to the Declaration of Helsinki and in line with the principles of Good Clinical Practice and Human Subject Protection. Prior to collecting data, ethical clearance was sought from the Research Ethics Committee of Cure Children’s Hospital of Uganda, approval number CUREC-2022-45. The health profession interns were informed that participation in the study was voluntary, and informed consent was sought.

## Results

### Sociodemographic characteristics of participants

A total of 281 participants (97% response rate) were recruited, with a mean (SD) age of 27 ± 3.8 years, the majority of which were male (*n* = 176; 62.6%) and doctors (*n* = 146; 52.0%). Most of the health profession interns had had prior training on AMR (*n* = 176; 62.6%) and used Medscape (*n* = 164; 58.4%) as their source of information, as summarized in Table 2.

**Table 2.** Sociodemographic characteristics and bivariate analysis of knowledge scores of participants (*N* = 281)

Variable	Frequency (%)	Knowledge score (mean ± SD)	Good knowledge		*P* value^a^
			Yes *n* (%)	No *n* (%)	
Overall		54.8 ± 22.7	53 (19)	228 (81)	
Age (mean ± SD)	27 ± 3.8				
Sex					
* *Male	176 (62.6)	58.3 ± 21.3	41 (77.4)	135 (59.2)	**0**.**014**
* *Female	105 (37.4)	48.9 ± 23.7	12 (22.6)	93 (40.8)	
Cadre of intern					
* *Doctor	146 (52.0)	57.1 ± 21.9	29 (54.7)	117 (51.3)	**0**.**048**
* *Pharmacist	65 (23.1)	64.5 ± 17.2	14 (26.4)	33 (14.5)	
* *Nurse	47 (16.7)	44.1 ± 25.8	9 (16.9)	56 (24.6)	
* *Midwife	23 (8.2)	50.7 ± 15.5	1 (1.9)	22 (9.7)	
Referral hospital					
* *Mulago	83 (29.5)	49.4 ± 21.4	11 (20.8)	72 (31.6)	0.087
* *Kiruddu	50 (17.8)	62.0 ± 21.0	12 (22.6)	38 (16.7)	
* *Kawempe	38 (13.5)	57.5 ± 20.8	8 (15.1)	30 (13.2)	
* *Mbarara	32 (11.4)	64.1 ± 16.5	8 (15.1)	24 (10.5)	
* *Mbale	50 (17.8)	44.0 ± 25.6	5 (9.4)	45 (19.7)	
* *Gulu	28 (9.9)	63.1 ± 21.9	9 (16.9)	19 (8.3)	
Prior AMR training					
* *Yes	176 (62.6)	54.2 ± 21.9	29 (54.7)	147 (64.5)	0.186
* *No	105 (37.4)	55.9 ± 24.0	24 (45.3)	81 (35.5)	
Source of information (Yes)					
* *UpToDate	118 (42.0)	54.7 ± 22.6	22 (41.5)	96 (42.1)	0.937
* *Medscape	164 (58.4)	58.5 ± 22.4	37 (69.8)	127 (55.7)	0.061
* *Hospital pharmacist	59 (21.0)	54.5 ± 22.7	9 (16.9)	50 (21.9)	0.426
* *Non-infectious disease physicians	25 (8.9)	56.7 ± 27.2	7 (13.2)	18 (7.9)	0.221
* *Infectious disease specialists	50 (17.8)	52.0 ± 26.9	12 (22.6)	38 (16.7)	0.306
* *Medical journals	109 (38.8)	58.1 ± 19.6	20 (37.7)	89 (39.0)	0.861
* *Peers (other interns)	76 (27.1)	55.3 ± 23.3	13 (24.5)	63 (27.6)	0.647
* *Guidelines by professional organizations	109 (38.8)	52.5 ± 21.5	28 (52.8)	81 (35.5)	**0**.**020**
* *Wikipedia	40 (14.2)	48.3 ± 23.5	3 (5.7)	37 (16.2)	0.050
* *Pharmaceutical representatives	48 (17.1)	62.1 ± 21.6	13 (24.5)	34 (14.9)	0.091
* *Others	26 (9.3)	56.4 ± 18.9	4 (7.5)	22 (9.7)	0.795

Bold type indicates statistical significance (*P* < 0.05).

a Chi-squared or Fisher’s exact tests performed.

### Knowledge on AMR and rational prescription practices

**Figure 1.**
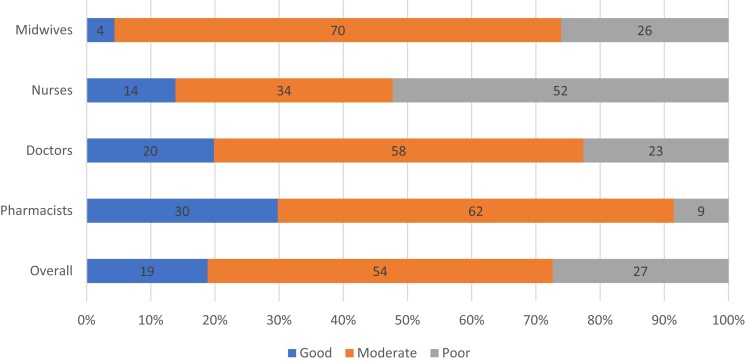
Categories of knowledge on AMR and rational prescription among participants overall and by different cadres.

Overall, few participants (*n* = 53; 19%) demonstrated good knowledge, while the majority had moderate (54%) or poor (27%) knowledge. Pharmacists had the highest percentage of good knowledge (*n* = 14; 30%), while nurses had the highest percentage of poor knowledge (*n* = 34; 52%), as shown in Figure 1. At bivariate analysis, factors correlated with good knowledge included the participant’s sex (*P* = 0.014), their cadre as an intern (*P* = 0.048) and whether they used professional organization guidelines as a source of information (*P* = 0.020) (Table 2). However, on multivariable logistic regression analysis, only the use of professional organization guidelines as a source of information was significantly associated with good knowledge (adjusted OR = 1.9; 95% CI: 1.0–3.5; *P* = 0.046), as shown in Table 3.

**Table 3.** Multivariable logistic regression of factors associated with good knowledge of AMR and rational prescription among participants

Variable	Adjusted OR	95% CI	*P* value
Sex			
* *Female	Reference		
* *Male	1.9	0.9–4.1	0.074
Cadre of intern			
* *Midwife	Reference		
* *Doctor	4.8	0.6–3.7	0.138
* *Pharmacist	7.3	0.8–4.0	0.065
* *Nurse	3.5	0.4–2.9	0.252
Guidelines by professional organizations			
* *No	Reference		
* *Yes	1.9	1.0–3.5	**0**.**046**

Bold type indicates statistical significance (*P* < 0.05).

The results showed that there was a significant difference in the mean knowledge scores among participants of different professional cadres (*P* < 0.0001), but not among those who had prior training on AMR versus those who did not (*P* = 0.554), as depicted in Figure S1 (available as Supplementary data at *JAC-AMR* Online). Specifically, the mean knowledge scores of doctors were significantly higher than those of nurses (57.11% versus 44.14%; *P* = 0.0005) and the mean knowledge scores of pharmacists were also significantly higher than those of nurses (64.55% versus 44.14%; *P* < 0.0001). However, there was no significant difference in the mean scores between doctors and pharmacists or between nurses and midwives, as shown in Table S1.

**Figure 2.**
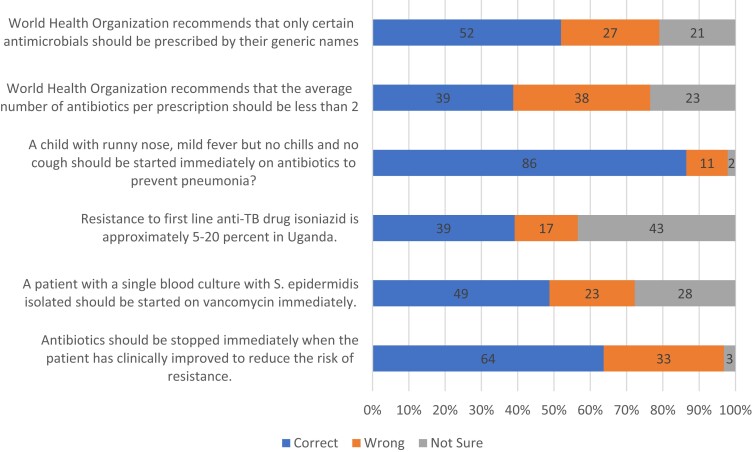
Responses of participants to knowledge questions on AMR and rational prescription.

Based on the clinical vignettes, most of the participants were aware that antimicrobials are not effective in treating the common cold (*n* = 243; 86%) and that complete dosage of antimicrobials should be taken despite early clinical improvement (*n* = 179; 64%), but only a few had knowledge about the national resistance patterns to the TB drug isoniazid (*n* = 110; 39%) and the WHO recommendations on the average number of antibiotics per prescription (*n* = 109; 39%). (Figure 2).

### Perception of AMR and rational prescription practices

**Figure 3.**
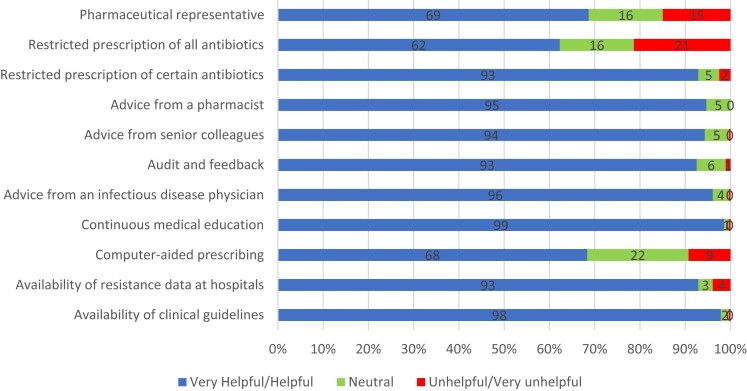
Perceptions of participants on the helpful interventions to combat AMR.

More than half of the study participants (*n* = 157; 56%) strongly believed that antimicrobials were overused in their hospitals and that excessive use of antimicrobials in livestock contributes to the spread of AMR (*n* = 133; 47%). Additionally, nearly half of the participants (*n* = 128; 46%) strongly agreed that prolonged antibiotic treatment durations are a significant factor in driving AMR, as summarized in Table 4. In terms of helpful interventions for combating AMR and improving prescriptions, the most commonly cited strategies included continuous medical education (CME, 99%), making clinical guidelines available (98%), seeking advice from infectious disease physicians (96%) and consulting with pharmacists (95%), as illustrated in Figure 3.

**Table 4.** Perceptions of participants towards AMR

Statement	SA *n* (%)	A *n* (%)	N *n* (%)	D *n* (%)	SD *n* (%)
Prescribing broad-spectrum antimicrobials when equally effective narrower-spectrum antimicrobials are available	120 (43)	83 (30)	8 (3)	39 (14)	31 (11)
Poor infection control practices by healthcare professionals	109 (39)	113 (40)	30 (11)	21 (7)	8 (3)
Excessive use of antimicrobials in livestock	133 (47)	72 (26)	28 (10)	30 (11)	18 (6)
Antimicrobial overuse at the hospitals	157 (56)	98 (35)	8 (3)	8 (3)	10 (4)
Too low doses of antibiotics	112 (40)	96 (34)	30 (11)	30 (11)	13 (5)
Too long durations of antibiotic treatments	128 (46)	100 (36)	25 (9)	15 (5)	13 (5)

SA, strongly agree; A, agree; N, neutral; D, disagree; SD, strongly disagree.

### Confidence level on prescribing antimicrobials

**Figure 4.**
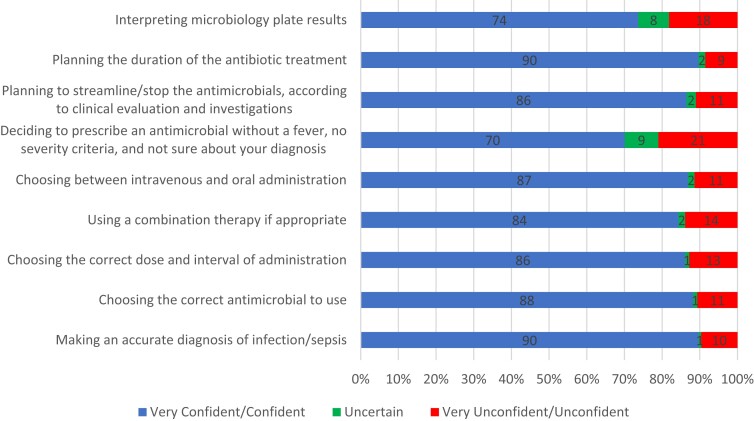
Confidence level of participants on antimicrobial prescription practices.

The majority of participants expressed confidence in their ability to make an accurate diagnosis of infection/sepsis (90%), choose the correct antimicrobial to use (88%), determine the correct dosage (86%), utilize combination therapy when appropriate (84%) and select the appropriate administration route (87%). However, participants were least certain about deciding whether or not to prescribe an antimicrobial in cases where the patient did not have a fever, exhibited no severity criteria, and had a confirmed diagnosis (21%), as depicted in Figure 4.

## Discussion

This study found that a small percentage (19%) of participants demonstrated good knowledge about AMR and rational prescription, while the majority had moderate or poor knowledge. This is consistent with similar studies done in Greece, France and Scotland among junior doctors.^14,15^ However, this is in contrast to a study carried out in Uganda, where almost 9 in 10 final-year clinical students had sufficient knowledge about AMR, especially those who had received prior training.^16^ This is possibly because the participants in the current study and those in Greece were mostly junior doctors who had been out of school for some time, while those in the later study were mostly medical students fresh with their knowledge of AMR in school. This highlights the importance of CME and creating stewardship programmes to keep healthcare professionals up to date on their knowledge. Indeed, almost all participants (99%) in the current study identified CME as the most helpful intervention.

The second most cited helpful intervention was availability of clinical guidelines (98%) and this study showed that using professional organization guidelines was the only factor independently associated with having good knowledge in consonance with a similar study in Italy.^17^ The reason for this is that professional organizational guidelines are based on carefully considered, approved, scientifically proven and current information. Another study in Ethiopia found that health profession interns who received antimicrobial stewardship training during the past 6 months had better attitudes and perceptions about AMR, further highlighting the need for refresher training.^9^ It is noteworthy that most junior doctors select antimicrobials based on practices of their older colleagues, who are liable to make errors as well, rather than on official guidelines.^12^ Therefore, emphasis should be placed on encouraging all levels of health workers to use standard guidelines.

We did not find any notable variation in the average scores between doctors and pharmacists, or between nurses and midwives. This contradicts a study conducted in three universities in East Africa, where pharmacy students had better knowledge about AMR and the usage of antibiotics in clinical scenarios compared with medical students, based on the average scores.^18^ However, nurses significantly had lower knowledge than doctors and pharmacists, similar to other studies.^19^ This is possibly because nurses and midwives typically administer drugs, but are seldom consulted when it comes to decisions about prescribing antibiotics. Clinical decisions about prescribing antimicrobials are typically made by doctors. It’s imperative that an interdisciplinary approach is adapted when dealing with antimicrobials.

The majority of the participants (86%) knew that antimicrobials are not effective in treating the common cold, similar to a study in Colombia^20^ but contrary to a study in the Democratic Republic of Congo, where more than two-thirds of the respondents would initiate antibiotic treatment,^21^ and another study in India, where 61.43% of undergraduate students, 74.29% of interns and 71.43% of postgraduate students incorrectly believed that antibiotics would hasten the recovery from cold and cough.^22^ Additionally, the participants demonstrated insufficient knowledge regarding questions regarding rational prescription guidelines established by the WHO in partnership with the International Network of Rational Use of Drugs (INRUD), based on questions evaluating this aspect,^23^ and the one on the national resistance patterns, which is publicly accessible from the National AMR Action Plan document. This emphasizes the need for junior doctors to be motivated to review guidelines established by their professional organization.

Nearly half of the participants (47%) held a strong conviction that the overuse of antimicrobials in livestock contributes to the proliferation of AMR, which is in agreement with the WHO’s assertion that the excessive and inappropriate utilization of antibiotics in both humans and animals is a significant factor contributing to the escalating risk of antibiotic resistance.^24^ The reckless use of antimicrobials in food processing and animal results in drug resistance, which endangers the health of both animals and humans alike.

Most of the participants expressed assurance in their ability to accurately diagnose infection/sepsis, determine the appropriate antimicrobial to use, ascertain the correct dosage, utilize combination therapy when suitable, and select the right administration route. This is different from a study conducted in Hong Kong, where interns had inadequate knowledge and low confidence, resulting in a restricted and passive role in prescribing, with a significant proportion compelled to follow the directions given by their superiors and influenced by senior nurses.^11^ However, participants were least certain about whether to prescribe antimicrobials in situations where the patient did not have a fever, showed no signs of severity, and had a confirmed diagnosis (21%). In some studies, health profession interns believe that the education they receive regarding appropriate antimicrobial use and stewardship is insufficient.^9^ It is essential to provide comprehensive, frequent and up-to-date training on antimicrobial stewardship and access to prescribing guidelines to guide the prescribing practices of health profession interns.

This study offers valuable insights into the knowledge, perceptions and confidence levels of diverse health profession backgrounds concerning AMR and rational prescription practices from regionally representative multiple centres in Uganda, which enhances the generalizability of the findings. The incorporation of various professional cadres of health profession interns adds a comprehensive dimension to the investigation, fostering a more holistic understanding of the topic. However, the study also faces some limitations. The reliance on self-reported data introduces the possibility of social desirability bias, especially on the confidence aspect, potentially affecting the accuracy of responses. The predominantly quantitative approach limits the exploration of contextual nuances that could enhance the interpretation of findings. Certain findings have been compared with those from studies that share some similarities but not complete congruence in participant inclusion, owing to the diverse range of participants in our investigation. While these comparisons still offer valuable insights that resonate with our study’s findings, their interpretation should be with caution.

### Conclusions

The study found that a majority of junior healthcare professionals have moderate to poor knowledge about AMR and rational prescription despite most participants being confident in their ability to accurately diagnose infections and sepsis and select appropriate antimicrobials. The disparity between junior healthcare professionals’ knowledge and their confidence suggests a need for further investigation into factors influencing this discrepancy. Future research could incorporate the objective measure of guideline compliance to gain insights into bridging this gap effectively. CME, stewardship programmes and availability of clinical and professional organization guidelines were identified as the most helpful interventions to improve this knowledge. The study also found that nurses had the lowest knowledge, indicating a need to improve their understanding of AMR. The participants had insufficient knowledge about rational prescription guidelines established by the WHO and the national resistance patterns. Therefore, the guidelines should not only be made available but also encourage junior health professionals to read and use them. The overuse of antimicrobials, both at the health facilities and in livestock, was recognized as a significant factor contributing to the escalating risk of antibiotic resistance. This is a call to the authorities and regulators to devise useful strategies to control the unnecessary use of antimicrobials.

## Supplementary Material

dlad105_Supplementary_Data

## Supplementary data

Figure S1 and Table S1 are available as Supplementary data at *JAC-AMR* Online.
