# Supplementary material for: Antimicrobial resistance and rational prescription practices: knowledge, perceptions and confidence of health profession interns in Uganda
Source: JAC Antimicrob Resist. 2023 Oct 3;5(5):dlad105. doi: 10.1093/jacamr/dlad105 (PMC10546811; doi:10.1093/jacamr/dlad105)
Supplement: dlad105_Supplementary_Data [file dlad105_supplementary_data.docx]

Table S1: Post Hoc Tukey HSD test on mean knowledge scores for comparison groups according to cadre of intern.

| **Comparison Group** | **Mean 1** | **Mean 2** | **Mean Difference** | **95% CI of difference** | **Adjusted P-Value** |
| --- | --- | --- | --- | --- | --- |
| Doctor versus Pharmacist | 57.11 | 64.55 | -7.444 | -16.87 to 1.985 | 0.1757 |
| Doctor versus Nurse | 57.11 | 44.14 | 12.97 | 4.588 to 21.35 | **0.0005*** |
| Doctor versus Midwife | 57.11 | 50.74 | 6.370 | -6.241 to 18.98 | 0.5601 |
| Pharmacists versus Nurse | 64.55 | 44.14 | 20.41 | 9.651 to 31.18 | **<0.0001*** |
| Pharmacist versus Midwife | 64.55 | 50.74 | 13.81 | -0.4917 to 28.12 | 0.0628 |
| Nurse verus Midwife | 44.14 | 50.74 | -6.601 | -20.24 to 7.039 | 0.5950 |
| *Statistically significant at p<0.05. | | | | | |

Figure S1: Comparison of mean knowledge scores among participants by (A) cadre and (B) prior training on AMR. Differences in mean knowledge scores across cadres was statistically significant (p<0.0001) unlike prior exposure to AMR training (p-0.554). a – One way ANOVA test performed, b – Independent t test performed.
